# Lung abscess secondary to lung cancer with *Eikenella corrodens* and *Streptococcus anginosus*: a case report

**DOI:** 10.1186/s12879-020-05054-y

**Published:** 2020-05-18

**Authors:** Leihao Hu, Jietao Lin, Jing Li, Yang Cao, Lizhu Lin

**Affiliations:** 1 First Clinical Medical College, Guangzhou University of Chinese Medicine, Guangzhou, Guangdong China; 2 Oncology Center, the First Affiliated Hospital of Guangzhou University of Chinese Medicine, Guangzhou, Guangdong China; 3 Guangzhou University of Chinese Medicine, Guangzhou, Guangdong China; 4 Oncology Department, the First Affiliated Hospital of Hunan University of Chinese Medicine, Changsha, Hunan China

**Keywords:** *Eikenella corrodens*, *Streptococcus anginosus*, Lung abscess, Lung cancer, Case report

## Abstract

**Background:**

*Eikenella corrodens* and *Streptococcus anginosus*, which are primary colonization bacteria of the normal flora of the oropharynx, are infrequent bacteria, especially the former. Here, we report a case of lung abscess with a coinfection of *Eikenella corrodens* and *Streptococcus anginosus* in a lung cancer patient.

**Case presentation:**

A 66-year-old Chinese man with lung cancer was admitted to the hospital, complaining of a cough and expectoration for five months and fever for two months. After a series of inspections to differentiate a cancer-related fever from an infectious fever, he was diagnosed with lung abscess. Draining pus culture demonstrated *Eikenella corrodens* and *Streptococcus anginosus*. After more than 1 month of antibiotic therapy and draining in total, he gradually recovered to fight against lung cancer.

**Conclusion:**

This report highlights the increased pathogenicity of *Eikenella corrodens* and *Streptococcus anginosus* in an immunocompromised cancer patient, especially after a few invasive operations. Additionally, even though a patient has been diagnosed with cancerous fever, strong vigilance is needed in case an infection arises.

## Background

In over 90% of cases of lung abscess, polymicrobial bacteria can be found. For decades, anaerobic bacteria have been the most dominant type of bacteria in lung abscesses, with *Streptococcus*, including *Streptococcus anginosus* [1]. *Eikenella corrodens*, also a kind of anaerobic bacteria, has been reported to cause lung abscess in only several cases [2–7]. *Eikenella corrodens* has been previously found to be associated with lung abscess in a lung cancer patient [8]. Coinfection with *Eikenella corrodens* and *Streptococcus* has been reported to cause abscesses in different body parts, including the liver, thigh and brain [9–11]. The two uncommon fastidious bacteria are both part of the normal microbiota of mucosa in humans [12, 13]. Sometimes, clinicians may not be able to easily differentiate a cancer-related fever and an infectious fever. Here, we report a case of lung abscess secondary to lung cancer with a coinfection of *Eikenella corrodens* and *Streptococcus anginosus*.

## Case presentation

A 66-year-old Chinese man was admitted to the Tumor Center of the First Affiliated Hospital of Guangzhou University of Chinese Medicine on September 21, 2018, complaining of a cough and expectoration for 5 months and fever for 2 months. During the period of visiting the outer hospital, chest and upper abdomen computed tomography (CT) scans were performed on April 26, showing a lung mass in the left inferior lung (size of approximately 45 × 43 mm) with some lymph node metastasis (including bilateral peribronchial lymph nodes, PBLN). CT-guided percutaneous biopsy and two endobronchial ultrasound-guided transbronchial needle aspiration (EBUS-TBNA) procedures were carried out for the biopsy in April. The pathology was adenocarcinoma. However, the patient refused to receive regular anticancer therapy until August. On August 1 and August 22, two courses of chemotherapy (tegafur gimeracil oteracil potassium capsule and carboplatin) were conducted, although low fever began in July. The prophylactic antibiotic was administered though the white blood cell count (WBCC), procalcitonin (PCT) and C-reactive protein (CRP) levels were in the normal range, after which there was defervescence. Based on his properties of fever, he was clinically diagnosed with cancer-related fever. In mid-September, the third chemotherapy was delayed to a vague later date because of a high fever, high WBCC and CRP levels, and severe myelosuppression when a chest CT showed a cyst cavity with the gas-liquid level in the left lung. Therefore, the patient was diagnosed with lung abscess and subsequently accepted antibiotic therapy and percutaneous abscess drainage. However, there was obstructed drainage and no symptom relief. The patient visited our oncology centre for further treatment.

**Fig. 1:**
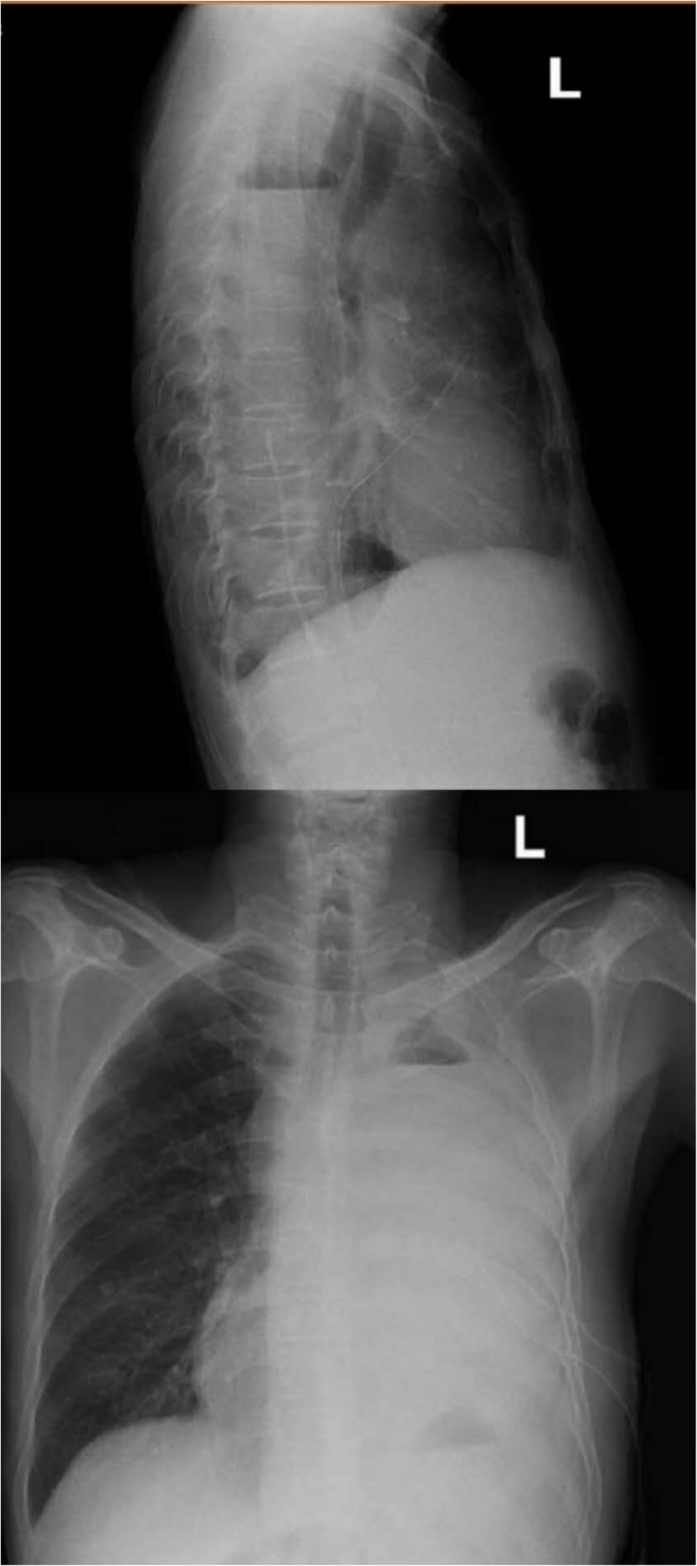
Chest X-ray image after the patient underwent closed thoracic drainage. It showed extensive hydropneumothorax (massive pleural effusion) with atelectasis

**Fig. 2:**
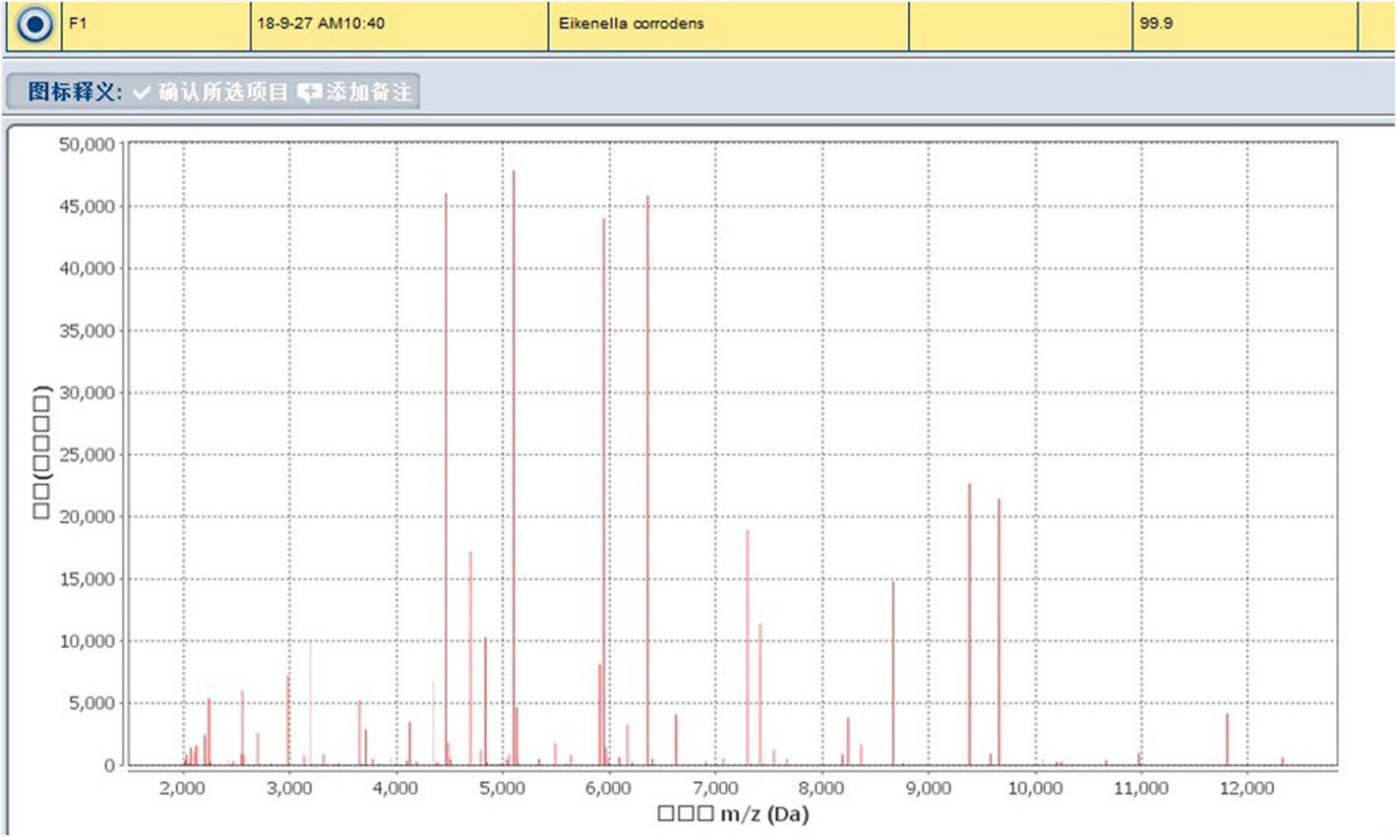
The outcomes of the microorganism mass spectrometer indicated that *Eikenella corrodens* and *Streptococcus anginosus* existed

**Fig. 3:**
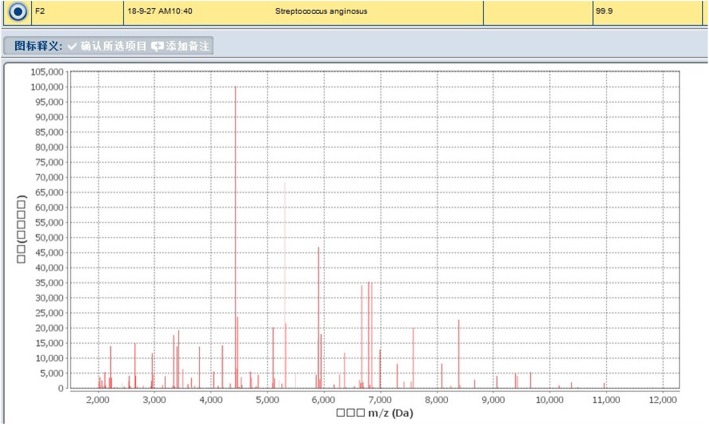
The outcomes of the microorganism mass spectrometer indicated that *Eikenella corrodens* and *Streptococcus anginosus* existed

**Fig. 4:**
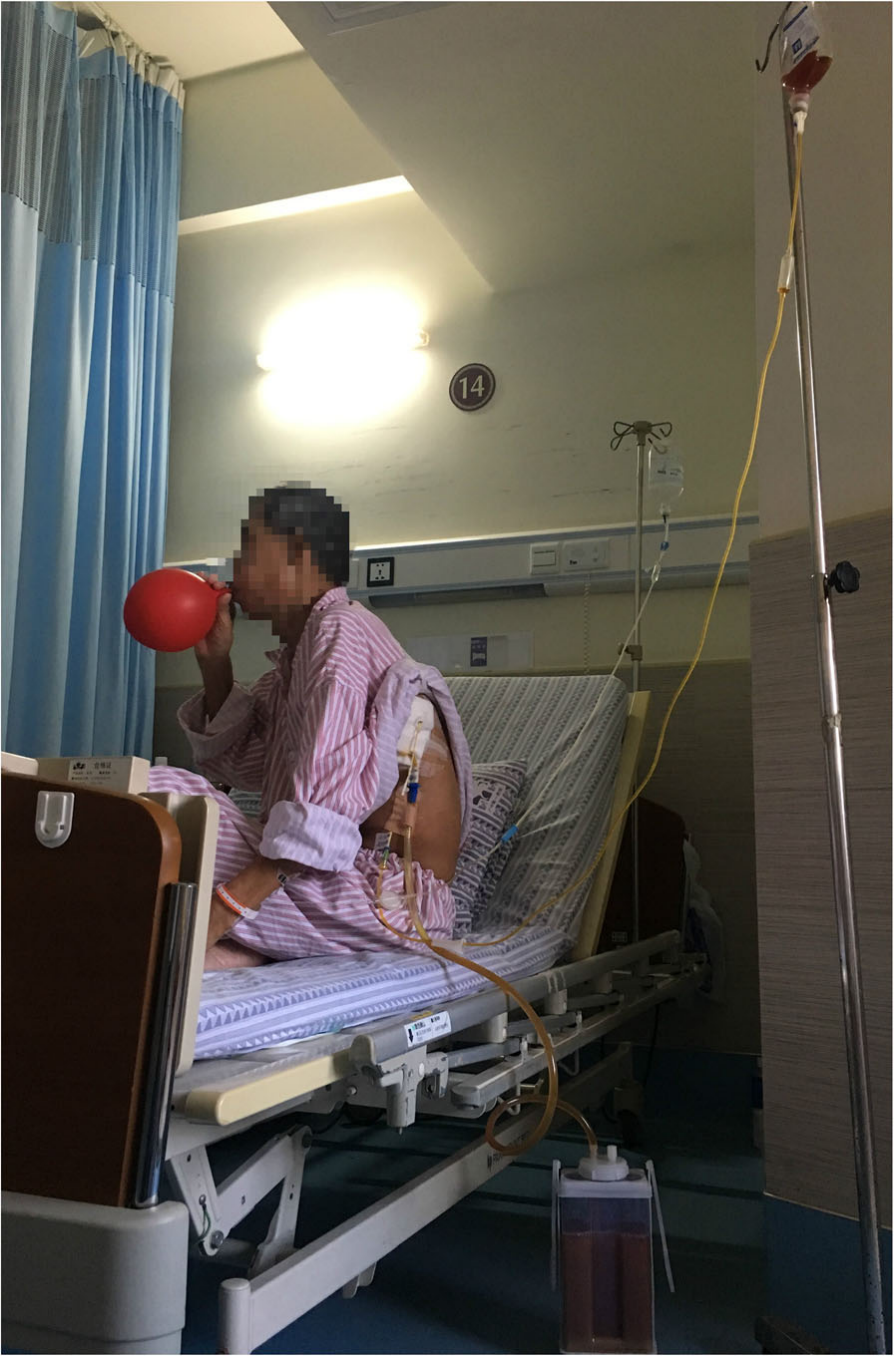
The patient was excepting thoracic washing with povidone iodine and metronidazole sodium chloride solution heated to physical temperature (for a total of 6 days, twice per day) and was encouraged to blow up balloons to enhance the draining

Once admitted, the patient underwent re-examination by CT, revealing extensive hydropneumothorax, and was then diagnosed with pyothorax. Moreover, laboratory inspection including high WBCC, CRP, PCT and low albumin levels, indicating infection and malnutrition. We adjusted the depth of the catheter that had been put in by the previous hospital and conducted closed thoracic drainage, after which the patient underwent a chest X-ray (Fig. 1). We empirically used intravenous moxifloxacin beginning on September 21. In addition, the blood culture results were negative. We ordered bacterial cultures four times for the draining pus, and each result was positive; the bacteria that grew included *Eikenella corrodens* and *Streptococcus anginosus* (Figs. 2, 3). There was no improvement in symptoms. Thus, on September 26, we added piperacillin-tazobactam with moxifloxacin to cover the pathogens. However, the response was not good until the bacterial susceptibility test was determined on September 28. According to the outcome, we changed piperacillin-tazobactam with cefoperazone sulbactam because the bacteria were resistant to penicillins. Concurrently, we began thoracic washing with povidone iodine and metronidazole sodium chloride solution heated to physical body temperature (for a total of 6 days, twice per day), and we encouraged him to blow up balloons to enhance the draining (Fig. 4). Echocardiography was performed on September 28, and the results showed that heart structure and function were both normal; therefore, we ruled out endocardial infection. Subsequently, the patient became afebrile, and the draining solution became cleared and tailed off. On October 18, the WBCC and CRP levels were in the normal range, and then he was discharged after more than 4 weeks of parenteral antibiotic therapy and thoracic draining.

Moreover, 7 days of oral moxifloxacin was prescribed as discharge medication. Nutrition support was run throughout the whole medication period. From a telephone follow-up in December, we were informed that the latest CT scan showed complete removal of the abscess and that he was undergoing further anti-tumour therapy.

## Discussion and conclusions

The aetiology, diagnosis and treatment options of lung abscess have been summarized [1]. Targeted antibiotics and thorough drainage are key to cure. As mentioned, with the extensive use of antibiotics, the mortality of lung abscesses has decreased to approximately 2–38.2% with the important roles of patient age, malnutrition, comorbidity, immunity, appropriate and timely antibiotics, and supportive therapy. Nevertheless, for a cancer patient, a lung abscess can be fatal because it worsens the patient’s condition and interferes with the anticancer treatment. Diagnosis and treatment must be determined as soon as possible to increase the time to fight against the cancer.

In the present case, contributing factors to lung abscess were elderly age, malnutrition, hypoimmunity due to the tumour and previous chemotherapy, bronchial obstruction related to the lung mass and enlarged PBLN, and multiple biopsies. *Eikenella corrodens* and *Streptococcus anginosus*, which are primary colonization bacteria of the mucosa, were isolated from the abscess. It is possible that the route of coinfection was via bronchi, and multiple biopsies, especially EBUS-TBNA, prompt the formation. There is a small chance that drainage may permit entry of the microorganisms into the cavity or haematogenous seeding.

Clinically, early signs and symptoms, including cough, expectoration, fever, weight loss, fatigue, chest pain, cannot effectively differentiate pneumonia, lung abscess, and lung cancer, which requires clinicians a have a keen observation for the condition. In this case, the expedited diagnosis and timely CT examination were the important aspects to differentiate a cancer-related fever from an infectious fever without delay. Thorough drainage, thoracic wash and rational choice of antibiotics give rise to recovery. Repetitive bacterial culture with pus even though the blood culture results were negative, which might be suppressed by antibiotics, helps us to obtain specific pathogens, which instructs us to use antibiotics. Notably, both *Eikenella corrodens*, a member of HACEK bacteria, and *Streptococcus anginosus*, a member of *Streptococcus viridans*, are significant infecting organisms for infective endocarditis [14]. Therefore, we performed echocardiography.

Overall, multiple invasive operations may enhance the possibility of opportunistic infection. To determine the pathogenic bacterium, the draining pus should be captured again despite the negative blood culture results. Even if *Eikenella corrodens* and *Streptococcus anginosus* have limited pathogenicity, they are potential pathogens for lung abscesses. The finding of *Eikenella corrodens*, a member of the HACEK group, and *Streptococcus anginosus*, a member of *Streptococcus viridans*, should cause observation of endocarditis even in patients without obvious symptoms of endocarditis. Moreover, clinicians need to pay attention to the prevention and differentiation of cancer-related and infectious fevers in cancer patients. The faster we make it clear, the more time we will buy for anticancer treatment.

## Data Availability

All the data and materials in this report are from the authors on reasonable request.

## Abbreviations

CT: Computed tomography
PBLN: Peribronchial lymph nodes
WBCC: White blood cell count
PCT: Procalcitonin
CPR: C-reactive protein
